# Canadian Physicians' Attitudes towards Accessing Mental Health Resources

**DOI:** 10.1155/2016/9850473

**Published:** 2016-04-10

**Authors:** Tariq M. Hassan, M. Selim Asmer, Nadeem Mazhar, Tariq Munshi, Tanya Tran, Dianne L. Groll

**Affiliations:** ^1^Providence Care Mental Health Services, 752 King Street West, Kingston, ON, Canada K7L 4X3; ^2^Department of Psychiatry, Queen's University at Kingston, ON, Canada; ^3^Queen's University at Kingston, ON, Canada

## Abstract

Despite their rigorous training, studies have shown that physicians experience higher rates of mental illness, substance abuse, and suicide compared to the general population. An online questionnaire was sent to a random sample of physicians across Canada to assess physicians' knowledge of the incidence of mental illness among physicians and their attitudes towards disclosure and treatment in a hypothetical situation where one developed a mental illness. We received 139 responses reflecting mostly primary care physicians and nonsurgical specialists. The majority of respondents underestimated the incidence of mental illness in physicians. The most important factors influencing respondent's will to disclose their illness included career implications, professional integrity, and social stigma. Preference for selecting mental health treatment services, as either outpatients or inpatients, was mostly influenced by quality of care and confidentiality, with lower importance of convenience and social stigma. Results from this study suggest that the attitudes of physicians towards becoming mentally ill are complex and may be affected by the individual's previous diagnosis of mental illness and the presence of a family member with a history of mental illness. Other factors include the individual's medical specialty and level of experience. As mental illness is common among physicians, one must be conscious of these when offering treatment options.

## 1. Introduction

In 2008, the WHO reported mental illnesses as the leading cause of disability in the US and Canada [1]. The incidence of mental illness among Canadians has been estimated at approximately 18.6% per year and has been shown to affect those of all genders, occupations, and sociocultural backgrounds [2, 3]. All considered, the impact of mental illness on the Canadian economy has totaled about $20.7 billion in 2012, and this number is expected to increase by 1.9% each year [4]. Many recent studies have shown that approaching this problem is not as simple as one would expect. Barriers to seeking care for mental illness in Canada include not only a lack of resources, but also stigma which makes patients reluctant to seek help [5].

In discussing the burden of mental illness among Canadians, the mental health needs of physicians are often overlooked. Due to recent changes in the healthcare landscape, physicians have an increasingly demanding task of excelling in clinical, academic, and managerial roles [6]. However, despite their rigorous training, studies have shown that physicians experience higher rates of mental illness, substance abuse, and suicide compared to the general population [7]. In one study, physicians were found to be at almost 6 times higher risk for suicide than the general population [8]. This increasingly evident problem has led the American Medical Association (AMA) to coin the term impaired physician as “one unable to fulfill professional or personal responsibilities because of psychiatric illness, alcoholism, or drug dependency” [9]. In addition to psychiatric illnesses, physicians have also been shown to suffer from* professional burnout*, a psychological “syndrome characterized by a loss of enthusiasm for work (emotional exhaustion), feelings of cynicism (depersonalization), and a low sense of personal accomplishment” [10]. In a recent study of US physicians, almost half reported at least one symptom of burnout [10]. Although not in itself a mental illness, burnout may “reduce the effectiveness and overall positive energy a physician may have on a daily basis” and has been shown to have a relationship with increased rates of physical illness, depression, and substance use [11–13]. Aside from its personal impact, decreased physician wellness has been linked to decreased quality of healthcare and poorer patient outcomes [14].

Physicians' attitudes towards illness and coping begin early in medical training. Medical students were found to be more likely to underestimate their risk for mental illness [15]. Another contributor was found to be students' lack of personal experience with mentally ill patients, which would make them uncertain or hesitant about recognizing signs of mental illness or knowing how to seek help. Within a culture where weakness is not tolerated and doctors are seen as being “invulnerable” to illness, young medical trainees felt that their distress was overlooked and that they were discouraged from seeking help [16]. This seems to be further fortified in postgraduate training, where surveyed residents continue to experience stigma, with fears of jeopardizing their training status by seeking help for illnesses [17]. This attitude tends to worsen as one enters practice, as taking care of oneself tends to be “low on a physician's list of priorities” [18], and a third of physicians do not have a family physician themselves [19].

Only a handful of studies have begun to look at the prevalence of mental health concerns among Canadian physicians. Data from the 2007-2008 Canadian Physician Study revealed nearly a fifth to quarter of physicians admitting to symptoms of depression and anhedonia. This seemed to be higher in females and family physicians. Contributing factors identified included stress and poor work-life balance. Most strikingly, more than a quarter of Canadian physicians reported “mental health concerns that made it difficult for them to handle their workload in the past month” [20].

Only recently have we begun to gain insight into the burden of mental health concerns among Canadian physicians. However, little is known about what proportion of these individuals seek help. Learning more about what keeps physicians from accessing mental health services is our goal for this study. Until recently, there has been little interest in exploring the factors that present barriers for physicians to seek mental healthcare in particular. For example, a recent study revealed that among a group of physicians in the UK, over 75% of participants cited stigma as a factor influencing them to withhold disclosure of their mental illness to a professional [21].

Although very limited data exists for some physician populations outside of Canada, our literature review revealed no published literature reflecting Canadian physicians' attitudes towards becoming mentally ill or having a mental illness.

The goal of the current study was to explore barriers impacting access to mental healthcare for Canadian physicians. In the hypothetical situation that one developed a mental illness, our questionnaire explored where one would seek advice and which factors would affect a physician's decision to select a specific outpatient or inpatient treatment facility. Furthermore, we hypothesized that there would be differences among these attitudes based on whether the physician had previously experienced a mental illness and had witnessed a family member suffer from a mental illness and/or their medical specialty and years of experience.

## 2. Methods

This is a random sample, cross-sectional study. Eligible participants included all physicians registered to practice in Canada. Residents and medical students were excluded. A random sample of 6000 physicians (approximately 10% of physicians listed) were selected from Scott's Canadian Medical Directory [22] and sent a letter of information regarding the study with a link to an online survey. A secure, online questionnaire using Fluid Surveys was used. Both English and French versions were deployed, and there was an option to change the language either at the beginning or in the middle of the survey. There was also a link to receive a paper survey with a prepaid envelope. The paper survey was identical to the online version in structure and content of questions. No identifying information was collected from subjects, and the authors had no way of correlating survey responses with individual subjects.

All subjects were eligible to enter a draw to win one of several $50 gift cards. To enter the draw, the subjects entered their name and address into a secure form. This data was used to identify the location of subjects, although this information was not linked to the original questionnaire. A letter with a link to a survey was sent to subjects and responses were collected from January 15 to April 30, 2015.

### 2.1. Ethical Consideration

Prior to commencement, ethical approval was granted from the Queen's University Health Sciences and Affiliated Teaching Hospitals Research Ethics Board.

### 2.2. Statistical Analysis

All survey responses were entered directly into the Fluid Survey site and then downloaded into an excel file where they were checked for completeness and then analyzed using SPSS (IBM Corp) v22. Descriptive variables such as frequency counts, percentages, means, and standard deviations were used to describe the study participants. A series of two-sample chi-square tests (*χ* ^2^) were conducted to examine differences in responses based on identifying information (i.e., years of experience and specialty type).

## 3. Results

### 3.1. Sample Characteristics

**Figure 1:**
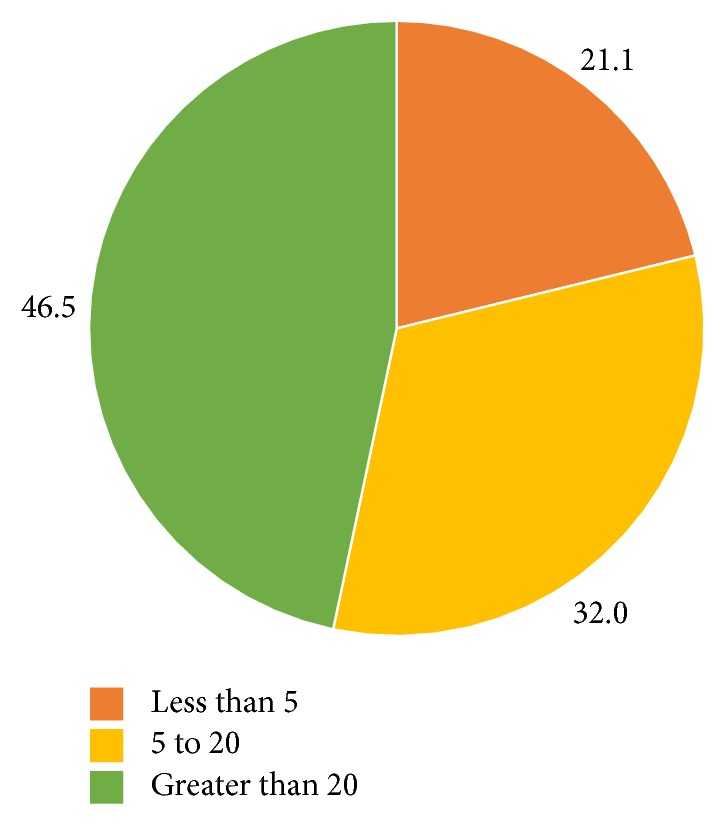
Canadian physicians divided by range of years they have been practicing. Data values represent frequencies in percentages (*N* = 128).

**Figure 2:**
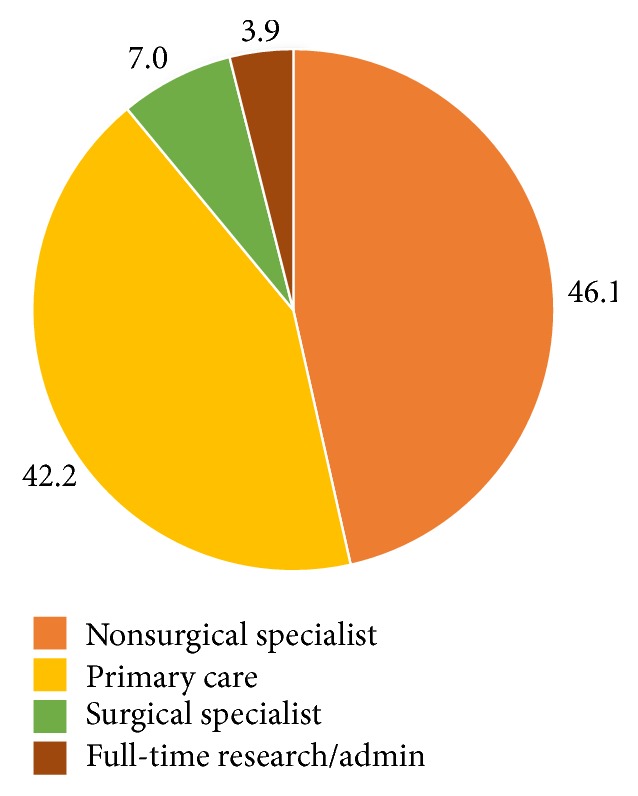
Canadian physicians divided by specialization. Data values represent frequencies in percentages (*N* = 127).

**Figure 3:**
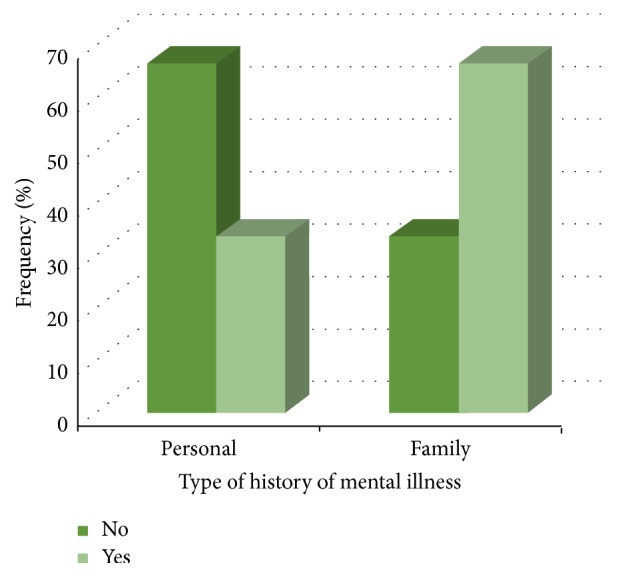
Frequency of Canadian physicians with or without personal and family history of mental illness (*N* = 128).

Of the 6000 questionnaires deployed, we received 137 responses (2.12% response rate). 92% of surveys were completed in English, and 8% were completed in French. Demographic information is summarized in Table 1. Respondents were mostly from Ontario (36%) and Quebec (19%), with lesser representations from all other Canadian provinces. Location information was missing for 18% of respondents, as they had not filled out their address for the draw. The greatest proportion of respondents were physicians with over 20 years of medical experience (46.5%), with a smaller proportion of physicians with 5–20 years of experience (32.0%) and 21.1% with less than 5 years of experience (Figure 1). Most respondents were characterized as being nonsurgical specialists (45.6%) and primary care physicians (41.9%) (Figure 2). The proportion of respondents reporting having had a personal history of mental illness was 31.6%. The proportion of respondents having had a family history of mental illness was 62.5% (Figure 3).

### 3.2. Estimates of Incidence of Mental Illness among Physicians

**Figure 4:**
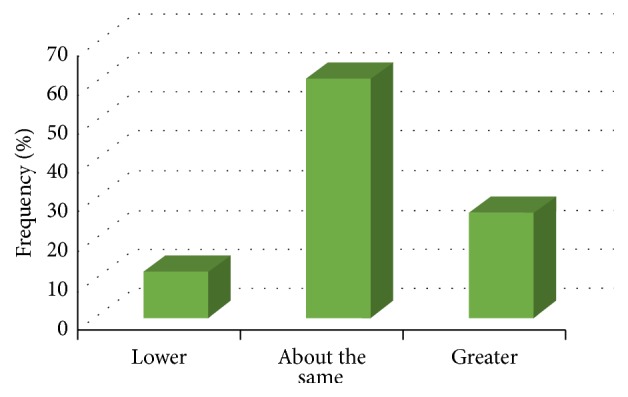
Canadian physicians divided by their estimation of the incidence of mental illness amongst physicians relative to the general population (*N* = 127).

Most respondents estimated the incidence of mental illness among physicians to be “about the same” as the general population (60.9%) (Figure 4). This estimate was significantly associated with family history of mental illness: *χ* ^2^  (*N* = 137) = 9.67,  *p* = 0.008, and Cramer's *V* = 0.28, with respondents who had a family history being more likely to respond as “about the same” or “higher” than the general population. Respondents with no family history of mental illness (23.8%) were more likely to incorrectly estimate the incidence as being lower than in the general population.

### 3.3. Disclosure of Mental Illness

The majority of participants (58.8%) preferred to disclose their mental illness to a family member, while much lower proportions of participants preferred to disclose their mental illness to their family physician (12.5%) or other healthcare professionals (9.6%). There was no correlation with varying levels of experience or past history of personal mental illness or family mental illness.

### 3.4. Seeking Advice for Management of Illness

Respondents were asked how they would seek advice in the hypothetical scenario where they would develop a mental illness affecting their functioning. As a first choice, respondents indicated a noticeably stronger preference towards formal outpatient advice (e.g., a referral through a family physician) (69.5%) compared to informal outpatient advice (e.g., advice from a friend or colleague) (18%). Self-medication (5.5%) or no treatment (3.1%) was least preferred (Table 2). There was no significant correlation with years of experience or personal and/or family history of mental illness.

### 3.5. Preferences with regard to Obtaining Treatment

Respondents were asked to select which factors would affect where they would seek outpatient care. The most important factors were quality of care (53.7%), followed by confidentiality (30.9%) (Table 3). Convenience and social stigma were least important, having been selected by 11.0% and 0.7% of respondents, respectively. Differences in years of experience or personal or family history of mental illness were not associated with different responses.

If inpatient treatment was to become necessary, no statistical differences were found regarding which facility type (government or private) or location (i.e., local or out-of-area facilities) respondents would choose (Table 4). There was no significant difference in response associated with different participant demographics. As with outpatient services, quality of care and confidentiality were the most important factors influencing facility selection (43.8% and 45.3%, resp.) (Table 5). Convenience and social stigma were the least important factors affecting facility selection (6.3% and 4.7%, resp.).

## 4. Discussion

### 4.1. Representativeness of the Study Sample

This is the first nationwide study in Canada exploring the attitudes of physicians towards becoming mentally ill and their preferences towards disclosing their mental illness, seeking treatment advice, and choosing a treatment facility. The response rate for this study was much lower for our survey was smaller than previous studies we have published [21, 23]. A strength of our study was that respondents reflected a variety of specialty types, mostly nonsurgical specialists and primary care physicians, which account for the biggest proportion of physicians in Canada. Surgical specialists were significantly underrepresented, possibly due to busier lifestyles and less free time to complete the survey. However, this data does not adequately represent Canadian physicians due to the low response rate and small sample size. The data suffered from undercoverage and nonresponse biases. The overrepresentation of physicians with a previous history of mental illness, as well as with family history of mental illness, indicated a source of voluntary response bias.

### 4.2. Understanding the Impact of Mental Illness

Over the past two decades, we have developed a greater understanding of physician mental illness, including its incidence, causes, and complications. One of the first questions in the survey explored knowledge of the prevalence of mental illness among physicians. Most participants indicated that the prevalence was the same as the general population, when in fact it is known to be higher. This indicates a need for physicians to be more educated during their training about how mental illness can affect them. More and more medical schools are promoting “wellness” and “resilience” as part of their medical school curricula, but these efforts are still in their infancy and are focused mostly on primary prevention. While these efforts are very effective in helping to improve rates of depression, anxiety, and burnout, there is currently little emphasis on secondary prevention and seeking help once a serious mental illness may be suspected [24, 25].

### 4.3. Seeking Advice: Has Stigma Improved?

When selecting which factors would affect their decision to disclose their mental illness and to seek help, social stigma was consistently one of the least selected factors. This is reassuring in the sense that it indicates that physicians understand the importance of formal mental healthcare in the treatment of psychiatric conditions and that they are not invulnerable. This is in stark contrast to previous studies in which physicians would much rather self-medicate than risk having their mental illness documented in their medical record [26]. Our results may suggest an overall trend towards decreasing stigma towards mental illness in today's doctors.

### 4.4. Quality of Care Is Most Important

The two most important factors identified in this study that influenced physicians' decision to select a treatment center, whether as inpatient or outpatient, were quality of care and confidentiality. This is consistent with our previous study of psychiatrists in Canada. As we move towards more centers using electronic patient records, physicians may be concerned about the confidentiality of their medical records. This may indicate a need for more education or modifications to existing physician healthcare programs to highlight both quality of care and confidentiality as part of their care services.

## 5. Conclusion

The results of our study indicate the decreasing importance of stigma in physicians' thinking regarding their own mental health concerns and the importance of quality of care and confidentiality in physicians seeking help. It remains important to continue advocating for the importance of mental health during medical training and educating physicians about the burden of mental illness in the physician population. Ongoing research is needed to further understand the factors that affect physicians' choices to seek treatment for their mental illness, as well as the effectiveness of education in affecting mental illness rates among physicians.

## Figures and Tables

**Table 1:** Demographics of survey respondents.

	*N*	%
Language		
English	118	92.2
French	10	7.8
Missing	—	—
Location		
Ontario	46	35.9
Quebec	24	18.8
British Columbia	10	7.8
Alberta	10	7.8
Nova Scotia	7	5.5
New Brunswick	2	1.6
Newfoundland and Labrador	2	1.6
Saskatchewan	2	1.6
Prince Edward Island	1	0.8
Missing	24	18.8
Practicing years		
Less than 5	27	21.1
5 to 20	41	32.0
Greater than 20	59	46.5
Missing	1	0.8
Specialization		
Nonsurgical specialist	59	46.1
Primary care	54	42.2
Surgical specialist	9	7.0
Full-time research/admin	5	3.9
Missing	1	0.8
Personal history of mental illness		
No	85	66.4
Yes	43	33.6
Missing	—	—
Family history of mental illness		
No	43	33.6
Yes	85	66.4
Missing	—	—

**Table 2:** Top picks for outpatient services.

Factor	Primary	Secondary	Tertiary
Formal	69.5	21.1	8.6
Informal	18	62.5	9.4
Self-medication	5.5	4.7	20.3
No treatment	3.1	2.3	21.1
Other	3.9	9.4	40.6

**Table 3:** Most important factors influencing top pick for outpatient treatment.

Factor	Frequency (%)
Quality of care	56.3
Confidentiality	32
Convenience	10.9
Social stigma	0.8

**Table 4:** Top picks for inpatient services.

Factor	Primary	Secondary	Tertiary
Local government facility	30.5	9.4	12.5
Out-of-area government facility	21.1	34.4	27.3
Local private facility	16.4	34.4	41.4
Out-of-area private facility	32	21.9	18.8

**Table 5:** Most important factor influencing top pick for inpatient treatment.

Factor	Frequency (%)
Quality of care	43.8
Confidentiality	45.3
Convenience	6.3
Social stigma	4.7
